# The Interaction between Human Microbes and Advanced Glycation End Products: The Role of *Klebsiella* X15 on Advanced Glycation End Products’ Degradation

**DOI:** 10.3390/nu16050754

**Published:** 2024-03-06

**Authors:** Aiying Shi, Xuemeng Ji, Wanhua Li, Lu Dong, Yuekun Wu, Yunhui Zhang, Xiaoxia Liu, Yan Zhang, Shuo Wang

**Affiliations:** Tianjin Key Laboratory of Food Science and Health, School of Medicine, Nankai University, Tianjin 300071, China; aiyingshi@mail.nankai.edu.cn (A.S.); jixuemeng@nankai.edu.cn (X.J.); wanhuali@mail.nankai.edu.cn (W.L.); donglu@nankai.edu.cn (L.D.); yuekunw@126.com (Y.W.); 2120201269@mail.nankai.edu.cn (Y.Z.); 1120200615@mail.nankai.edu.cn (X.L.); wangshuo@nankai.edu.cn (S.W.)

**Keywords:** advanced glycation end products, human gut microbiota, *Klebsiella*, proteomics

## Abstract

Previous studies have shown that advanced glycation end products (AGEs) are implicated in the occurrence and progression of numerous diseases, with dietary AGEs being particularly associated with intestinal disorders. In this study, methylglyoxal-beta-lactoglobulin AGEs (MGO-β-LG AGEs) were utilized as the exclusive nitrogen source to investigate the interaction between protein-bound AGEs and human gut microbiota. The high-resolution mass spectrometry analysis of alterations in peptides containing AGEs within metabolites before and after fermentation elucidated the capacity of intestinal microorganisms to enzymatically hydrolyze long-chain AGEs into short-chain counterparts. The 16S rRNA sequencing revealed *Klebsiella*, *Lactobacillus*, *Escherichia-Shigella*, and other genera as dominant microbiota at different fermentation times. A total of 187 potential strains of AGE-metabolizing bacteria were isolated from the fermentation broth at various time points. Notably, one strain of *Klebsiella* exhibited the most robust growth capacity when AGEs served as the sole nitrogen source. Subsequently, proteomics was employed to compare the changes in protein levels of *Klebsiella* X15 following cultivation in unmodified proteins and proteins modified with AGEs. This analysis unveiled a remodeled amino acid and energy metabolism pathway in *Klebsiella* in response to AGEs, indicating that *Klebsiella* may possess a metabolic pathway specifically tailored to AGEs. This study found that fermenting AGEs in healthy human intestinal microbiota altered the bacterial microbiota structure, especially by increasing *Klebsiella* proliferation, which could be a key factor in AGEs’ role in causing diseases, particularly intestinal inflammation.

## 1. Introduction

Advanced glycation end products (AGEs) are formed through the Maillard reaction, a non-enzymatic process occurring between free carbonyl and amino groups in proteins, peptides, or amino acids [1]. Numerous studies have demonstrated that endogenous AGEs can inflict harm upon the body [2]. Recent research has further revealed the role of dietary AGEs in the initiation and progression of numerous diseases, including non-alcoholic fatty liver disease [3], diabetes and its complications [4], and Alzheimer’s disease [5], among others. They achieve this by cross-linking with proteins [6], thereby altering their physiological functions, or by binding to receptors and activating signaling pathways, such as inflammation [7].

AGEs primarily form through the Maillard reaction during thermal processing. Foods abundant in both protein and sugar are prone to AGE formation when subjected to heating; examples include bread, barbecued meats, and margarine. The formation of AGEs involves the modification of lysine and arginine residues within proteins [8]. This alteration effectively occludes the trypsin digestion sites, thereby impeding the gastrointestinal breakdown of bound AGEs upon ingestion [9]. Kinetic assessments have revealed that a mere 10–30% of dietary AGEs are absorbed by the human body [10], with the majority excreted in feces through the colon. Recent studies have indicated that unabsorbed AGEs can influence the human body by interacting with intestinal microorganisms [11]. In a specific study, adolescent males subjected to a two-week high-AGE diet intervention exhibited quantitative changes in seven bacterial species in their fecal microbiota [12]. A high-AGE diet was correlated with an increase in Enterobacteriaceae. Additionally, investigations have demonstrated that dietary AGEs lead to a reduction in *Prevotella copri* and *Bifidobacterium animalis*, along with an increase in *Alistipes indistinctus*, *Clostridium hathewayi*, *Clostridium citroniae*, and *Ruminococcus gauvreauii* [13]. Animal experiments have further illuminated the impact of AGE-rich diets on the gut microbiota. These studies have revealed a reduction in α-diversity among mice subjected to heat-treated diets [14]. Moreover, heat-treated diets have been associated with a decrease in the abundance of Bacteroidetes and an increase in the abundance of Firmicutes. Our prior study revealed a correlation between a high-AGE diet and induced intestinal inflammation in mice, closely tied to changes in intestinal microbiota [15]. These findings emphasize that a diet abundant in protein-bound AGEs can indeed induce substantial structural alterations in the composition of the intestinal microbiota.

Over the past decade, the bidirectional interaction between AGEs and the gut microbiota has attracted research attention. During an in vitro experimental study, carboxymethyllysine (CML) was co-cultured with six strains of *Escherichia coli* (*E. coli*), and it was observed that five of these *E. coli* strains could effectively degrade free CML under aerobic conditions [16]. Furthermore, when CML was conjugated with dipeptides and co-cultured with *E. coli*, the metabolic activity was notably enhanced. It was discovered that microorganisms in the fecal samples of two volunteers exhibited the capability to metabolize 77% and 100% of free CML, respectively [17], confirming their ability to metabolize free CML under hypoxic conditions. It is important to note that previous studies have primarily focused on free AGEs. Nevertheless, AGEs consumed through dietary sources are predominantly protein-bound AGEs. And it is conceivable that the intestinal microbiota can directly interact with dietary AGEs, suggesting that the intestinal microbiota can metabolize AGEs. This ability to metabolize AGEs may confer advantages to individuals harboring specific bacterial strains proficient in AGE metabolism. However, specific strains involved in the degradation of AGEs and their key metabolic enzymes remain unclear. Previous studies have extensively explored the microbial metabolism of glycated proteins. However, the majority of these studies have centered on complex food matrices or mixtures of proteins, with a paucity of research specifically dedicated to individual proteins within defined conditions. Simultaneously, the emphasis has predominantly centered on the collective intestinal microbiota, leaving a gap in research on the mechanisms involving specific microbial strains in metabolizing protein-bound AGEs.

Our research aims to reveal shifts in gut microbiota composition and modifications in methylglyoxal-beta-lactoglobulin AGEs (MGO-β-LG AGEs) during fermentation, providing insights into the interaction between human intestinal microbiota and protein-bound AGEs. Assuming the presence of bacteria capable of autonomously metabolizing protein-bound AGEs, they could be isolated from fecal microbiota. Subsequently, proteomics methods could be utilized to analyze the process of metabolizing protein-bound AGEs. This discovery has the potential to broaden our comprehension of the bacterial hydrolysis of AGEs and offer valuable insights into the intricate interactions between gut microbiota and protein-bound AGEs.

## 2. Materials and Methods

### 2.1. Materials and Reagents

Bovine β-LG and methyglyoxal (40% aqueous solution) were obtained from Sigma-Aldrich (St. Louis, MO, USA). CML and carboxyethyllysine (CEL) (98%, HPLC) were obtained from Toronto Research Chemicals (Toronto, ON, Canada). Both pepsin and trypsin were sourced from Sigma-Aldrich (St. Louis, MO, USA). BHI medium was acquired from Thermo Fisher (Waltham, MA, USA). Acetonitrile and methanol for mass spectrometry were purchased from Merck (Darmstadt, Germany). Milli-Q water was employed in the experiments. All other reagents used were of analytical grade.

### 2.2. Preparation of MGO-β-LG AGEs

MGO-β-LG AGEs were prepared following our previous study [18]. β-LG and methylglyoxal were subjected to heating in a phosphate buffer (PBS, 0.05 M, pH 7.0) at 100 °C for 1 h. This entire process was conducted within a metal bath, ensuring that the liquid level in the reaction vessel did not surpass the level of the metal block. Subsequently, the heated mixture underwent dialysis using a dialysis bag, with the process carried out at 4 °C for 72 h. This was performed to eliminate any excess small molecule substances from the system. The resulting solution was then pre-frozen at −80 °C and subsequently lyophilized to yield MGO-β-LG AGEs in powdered form. The obtained powder was stored at −20 °C until further use.

### 2.3. In Vitro Gastrointestinal Digestion of MGO-β-LG AGEs

Following established research protocols [19], an in vitro digestion model system was performed to digest MGO-β-LG AGEs, a process consisting of two primary stages: gastric digestion and intestinal digestion. Before digestion, enzyme activity was quantified. The pH of the gastric buffer stock solution (1.25×, see Table S1) was adjusted to pH 3.0 using 0.5 M hydrochloric acid. MGO-β-LG AGEs (4 g/L) were introduced, along with the addition of pepsin to achieve a concentration of 2000 U/mL, and CaCl_2_ solution to reach a final concentration of 0.075 mM. The pH was subsequently readjusted to 3.0, and the volume was set with Milli-Q water. This mixture underwent a 2 h incubation at 37 °C with constant agitation at 100 rpm. Subsequently, the gastric digestive juice, resulting from the aforementioned reaction, was introduced into the intestinal buffer stock solution (1.25×, Table S1). This was performed to establish a final volume ratio of simulated gastric and simulated intestinal digestive juices at 1:1, with pH adjustment to 7.0 using 1 M NaOH. Then, trypsin was introduced, achieving a final concentration of 100 U/mL. Additionally, bile salts were added to attain a final concentration of 10 mM, along with CaCl_2_ solution at a final concentration of 0.3 mM. The pH was readjusted to 7.0, followed by volume adjustment using Milli-Q water. Subsequent incubation took place in a constant temperature shaker at 37 °C for 2 h, and to terminate the reaction the mixture was heated at 100 °C for 3 min.

### 2.4. Anaerobic Incubations of Fecal Slurries with Digested MGO-β-LG AGEs

All volunteers provided written informed consent before they participated in the experiment. Fresh fecal samples were collected from a total of 12 volunteers, comprising 5 men and 7 women, aged between 21 and 30 years old. None of the participants were pregnant, had gastrointestinal diseases, or had used antibiotics within three months before sample collection. It is important to note that all volunteers gave explicit informed consent before contributing their samples. For the preparation of the anaerobic bicarbonate buffer, the composition was as follows (in mM): NaHCO_3_, 47.62; Na_2_HPO_4_·2H_2_O, 2.98; KH_2_PO_4_, 3.01; CaCl_2_·2H_2_O, 0.75; MgCl_2_·6H_2_O, 0.49; NaCl, 5.13; Na_2_S·9H_2_O, 2.00 [17]. The culture medium was then prepared using MGO-β-LG AGEs at a concentration of 4 g/L as the sole nitrogen source. Additionally, glucose was added at a concentration of 2 g/L. The prepared bicarbonate buffer solution was aliquoted into specialized rolling tubes, and nitrogen gas was introduced to remove oxygen from the bottles. The tubes were then sealed and sterilized using high-pressure steam (121 °C, 15 min). After cooling, MGO-β-LG AGEs freeze-dried powder was added. Simultaneously, 0.1% *v*/*v* of an acidic trace element mixed solution was introduced with the following composition (in mM): FeCl_2_, 7.5; H_3_B0_4_, 1; ZnCl_2_, 0.5; CuCl_2_, 0.1; MnCl_2_, 0.5; CoCl_2_, 0.5; NiCl_2_ 0.1; and HCl, 50. Furthermore, 0.1% *v*/*v* of an alkaline trace element mixed solution was added, consisting of (in mM) Na_2_SeO_3_, 0.1; Na_2_WO_4_, 0.1; Na_2_MoO_4_, 0.1; and NaOH, 10. Finally, 0.2% *v*/*v* of a vitamin mixed solution was included, with the following composition (in mM): biotin, 0.02; niacin, 0.2; vitamin B_6_, 0.5; riboflavin, 0.1; thiamine, 0.2; vitamin B_12_, 0.1; para-aminobenzoic acid, 0.1; vitamin B_5_, 0.1. All solutions were filtered through a 0.22 μm sterile filter before use. To process each fecal sample, 1 g of the sample was weighed, and 1 mL of sterile PBS solution was added. The suspensions from all 12 fecal samples were mixed, and filtered through sterile gauze to remove any residue, and 200 μL of the resultant mixed fecal suspension was introduced via a syringe into the prepared culture medium (15 mL). The Hungate roller tubes were placed in an anaerobic culture box (Mitsubishi, Tokyo, Japan) and added to anaerobic bags (Mitsubishi, Tokyo, Japan). The fermentation process was carried out in a shaker at 37 °C. Samples were extracted every 8 h from the initiation of fermentation and subsequently stored at −80 °C for future use.

### 2.5. Gut Microbiota Analysis during In Vitro Fermentation

The method details are described in the Supplementary Materials. Sequencing data, including all field and incubated samples, have been submitted to the NCBI Sequence Read Archive (SRP484236).

### 2.6. Isolation of Bacteria from Fermentation Broth

Samples collected both at 8 h of fermentation and at the conclusion of fermentation were cultured on Brain Heart Infusion (BHI) agar plates to isolate bacteria at various fermentation time points. These agar plates were subsequently incubated at 37 °C in an anaerobic environment within an anaerobic workstation for at least 48 h. Isolated colonies with distinct morphologies were selected from the plates and transferred to new BHI agar plates, a process repeated three times. The final single colony from the last plate was inoculated into liquid BHI for further cultivation. Then, 1 mL of the cultured bacterial liquid was harvested through centrifugation, and DNA extraction was performed following the instructions provided by the DNA extraction kit. The specific identification methods are described in the Supplementary Materials.

### 2.7. Analysis of CML and CEL by HPLC-MS/MS

The sample pretreatment procedure was initiated with the reduction of the sample. Initially, 4 mg of freeze-dried powder was weighed, followed by the addition of 2 mL of borate buffer (sodium tetraborate, 0.2 M, pH = 9.18). Simultaneously, 0.4 mL of sodium borohydride NaOH solution (sodium borohydride, 2 M and NaOH, 0.1 M) was introduced into the mixture. The reduction process was carried out at room temperature and allowed to proceed for 4–6 h. Upon completion of the reduction step, an equal volume of 12 M HCl was incorporated into the solution, resulting in a final concentration of 6 M. Subsequently, acid hydrolysis was performed in a 110 °C metal bath for 24 h. Following acid hydrolysis, purification was conducted using Oasis MCX 3cc LP Extraction Cartridges (60 mg/3 mL, Agela Technologies, Tianjin, China), after which the sample was prepared for subsequent detection.

The detection method used in this study was adapted from previous studies as follows: a liquid chromatography tandem mass spectrometry system manufactured by Waters (Milford, CT, USA) was employed for quantifying the content of CML and CEL. The mass spectrometer, Xevo Micro TQ-XS, was operated in ESI mode with positive electrospray and utilized Multiple Reaction Monitoring (MRM). Chromatographic separation was carried out on an Atlant HILIC Silica column (2.1 × 150 mm, 3 μm; Waters) maintained at a column temperature of 35 °C. Mobile phase A consisted of acetonitrile, while mobile phase B was a 0.1% formic acid aqueous solution. The flow rate, following the gradient program, was set at 0.4 mL/min. The elution program utilizing mobile phase A progressed as follows: 0–3 min, 80–50%; 3–6 min, 50%; 6–6.1 min, 50–80%; and 6.1–12 min, 80%. The injection volume was 10 μL, and the flow rate during the analysis was maintained at 0.2 mL/min. For detailed information concerning characteristic ions of CML and CEL, please refer to the Supplementary Materials.

### 2.8. Analysis of Modification of Digested MGO-β-LG AGEs Pre- and Host Fermentation by High-Resolution Mass Spectrometry

The FASP method was employed with minor adjustments for sample preparation, as follows. Fermentation broth from various stages was introduced into a pre-treated ultrafiltration tube (10 KDa, Thermo Fisher, Waltham, MA, USA) and centrifuged at 12,000× *g* for 20 min. The filtrate was discarded, and the retained material was washed four times with a 6 M guanidine hydrochloride solution. Subsequently, a reduction step was initiated using DTT (10 mM) at 50 °C for 30 min. This was followed by alkylation with TCEP (50 mM) at room temperature, with shaking at 500 rpm for 40 min. After five washes with 50 mM ammonium bicarbonate, trypsin (Promega (USA) Biotech Co., Ltd., Madison, WI, USA) was added for enzymatic hydrolysis at 37 °C for 16 h. The resulting filtrate was collected through centrifugation. A desalting column was employed for desalination, and the sample was freeze-dried for future use.

High-performance liquid chromatography was performed with an EASY-nLC™ 1200 liquid chromatograph (Thermo Fisher, Waltham, MA, USA) equipped with a C18 column (3 μm pore size, 75 mm i.d., 15 cm length, 100 Å). Mass spectrometry was conducted using an Orbitrap Fusion Lumos mass spectrometer (Thermo Fisher, Waltham, MA, USA) equipped with a nanoliter ion source. The ion spray voltage was set at 2.1 kV, and the capillary temperature was maintained at 320 °C. Mass spectra were acquired in data-dependent mode, with MS1 and MS2 spectra obtained through full scan mass spectrometry in positive ion mode. The Orbitrap mass analyzer boasted a primary resolution of 120,000 (at *m*/*z* 200) and a secondary resolution (at *m*/*z* 200). The precursor ion scanning range spanned from *m*/*z* 350 to 1550, while the product ion scanning range initiated at *m*/*z* 110. Simultaneously, the top 20 ions in the signal were selected for MS2 analysis, with a dynamic exclusion time of 10 s. A consistent amount of iRT (Biognosys) peptide was introduced into each sample during MS data acquisition. DDA raw data were processed using Proteome Discover 2.1 (Thermo Fisher, Waltham, MA, USA), and potential modifications were assessed using PMI-Byonic software (version 3.7), as illustrated in Table 1. This analysis primarily targeted peptides containing 4–20 amino acids. β-LG, with its 16 lysine (K) and 3 arginine (R) modification sites, exhibited AGE modifications at K24, K30, K63, K76, K85, K86, K91, K116, K117, K151, K157, R56, R140, and R164.

### 2.9. Proteomic Analysis of Klebsiella X15 Responses to Sole Utilization of AGEs as Nitrogen Source

The isolated strains were cultured anaerobically utilizing digested MGO-β-LG AGEs as the sole nitrogen source, with the medium composition detailed in Section 2.4. As a control, unprocessed β-LG protein was also employed. The bacterial pellet was harvested via centrifugation at −80 °C. Total bacterial protein was then extracted by adding B-per extraction solution (5 μL of B-per solution per milligram of bacterial pellet, Thermo Fisher, Waltham, MA, USA), and protease inhibitors and phosphatase inhibitors (Thermo, Waltham, MA, USA) were added before experimentation. The bacterial pellet was gently shaken with the B-per solution for 1 h at room temperature, followed by centrifugation at 8000× *g* for 20 min to collect the supernatant.

High-resolution mass spectrometry was employed to assess variations in bacterial protein levels when utilizing MGO-β-LG AGEs as the only nitrogen source. The sample pretreatment is detailed in Section 2.8. A Q Exactive Plus mass spectrometer coupled with an EASY nano Liquid Chromatography system (EASY nLC 1200, Thermo Fisher, Waltham, MA, USA) and an EASY nano inductive spray interface were utilized for analysis. The methodology drew upon previous research and involved the use of a Thermo Fisher Acclaim Pepmap nano-trap column (C18, 5 μm, 100 Å, 100 μm × 2 cm) and a Thermo Fisher EASY-Spray column (Pepmap RSLC, C18, 2 μm, 100 Å, 50 μm × 15 cm). The mobile phase for nano-liquid chromatography comprised 0.1% formic acid solution as phase A and 80% acetonitrile/0.1% formic acid as phase B. The gradient program for mobile phase B was as follows: 0–8%, 3 min; 8–28%, 42 min; 28–38%, 5 min; and 38–100%, 10 min. MS and MS/MS data were acquired in data-dependent mode. Mass spectrometry data were subsequently analyzed using MaxQuant (version 2.0.3.1) proteomics analysis software. The mass spectrometry proteomics data have been deposited to the Proteome Xchange Consortium (https://proteomecentral.proteomexchange.org, accessed on 17 February 2024) via the iProX partner repository [20,21] with the dataset identifier PXD048590.

### 2.10. Data Analysis

The experimental results represent the mean ± SEM obtained from a minimum of three independent experiments. Statistical analysis was conducted using a two-tailed Student’s *t*-test. Gene Ontology (GO) and KEGG Enrichment Analysis (KEGG) were annotated using the Uniprot database, and enrichment analysis was carried out using Cluster Profiler in the R programming language. Moreover, a protein interaction analysis was conducted using the STRING database (https://string-db.org/, accessed on 26 September 2023), a valuable tool for retrieving information on protein–protein interactions. When parameters exhibited a normal distribution, differences were assessed using one-way analysis of variance (ANOVA). Throughout all experiments, a significance threshold of *p* < 0.05 was applied to determine statistical significance.

## 3. Results

### 3.1. Changes in Gut Microbial Community during In Vitro Fecal Fermentation

**Figure 1:**
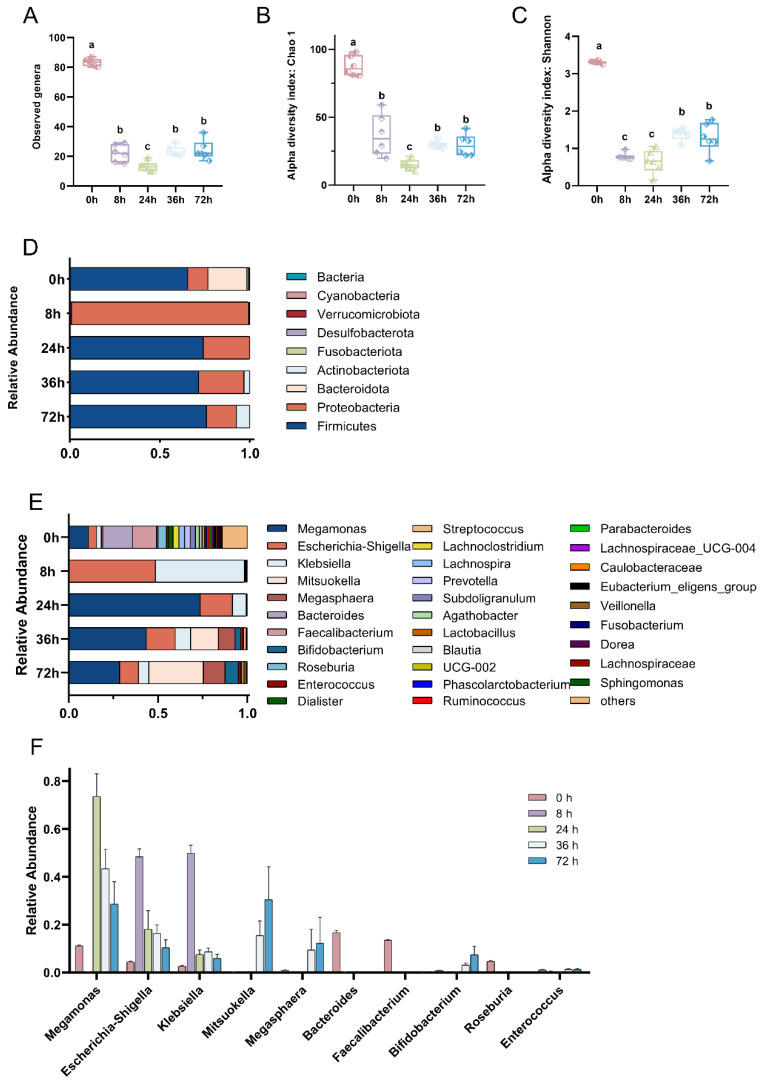
Dynamic evolution of gut microbiota composition across various fermentation time points. Comparison of microbial diversity at the different fermentation points by α-diversity analysis. Species richness and diversity measured by observed genus (**A**), Chao 1 (**B**), and Shannon diversity (**C**) at different fermentation points. Bar plots of relative abundance on phylum and genus levels (**D**,**E**). Top 10 bacterial genera in species abundance (**F**). In the figure, different lowercase letters represent significant differences between groups, with *p* < 0.05.

Throughout the in vitro fermentation process with digested MGO-β-LG AGEs as the only nitrogen source, the bacterial community composition exhibited temporal changes. The observed genera, Chao index, and Shannon index initially exhibited a notable decrease, followed by a subsequent increase (Figure 1A–C). The composition of the bacterial community at the phylum level is depicted in Figure 1D. In the initial microbial community, a balance was maintained among Bacteroidetes, Firmicutes, and Proteobacteria. However, subsequent to exposure to AGEs, the relative abundance of Bacteroidetes experienced a rapid decrease, surpassing a tenfold reduction at 8 h, with recovery not evident until the 72-h. The abundance of Firmicutes and Actinobacteria exhibited irregular fluctuations over the 72 h period. Proteobacteria displayed a rapid shift in relative abundance upon exposure to AGEs, constituting almost 100% at 8 h. This heightened relative abundance persisted above the initial levels until the 72nd hour, suggesting that Proteobacteria can selectively utilize AGEs to foster proliferation. 

At the genus level (Figure 1E), for the initial observation (0 h), a total of nine dominant bacterial genera were identified. However, by 8 h, the count of dominant bacterial genera sharply decreased to only two genera, namely *Klebsiella* and *Escherichia-Shigella.* Between 24 h and 72 h, there was a partial recovery in the number of dominant bacterial genera. Nevertheless, notable changes occurred in the types and abundances of these genera compared to the initial observation. This included an elevation in the relative abundance of *Megamonas*, *Escherichia-Shigella*, and *Klebsiella*. Additionally, *Mitsuokella* and *Megasphaera* emerged as new dominant bacterial genera.

**Figure 2:**
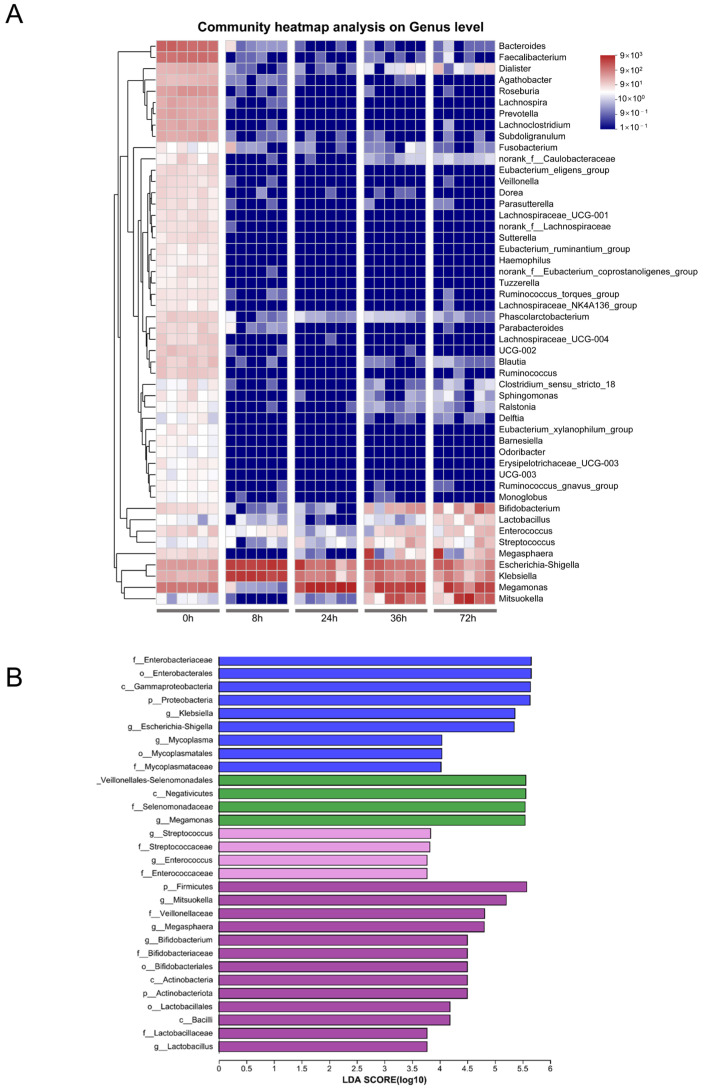
Heatmap and LEfSe analysis of gut microbiota. (**A**) Community heatmap analysis at genus level. (**B**) LEfSe comparison of gut microbiota; the species with an LDA score > 3.5 represent the statistically significant biomarkers among groups.

LEfSe and heatmap analysis revealed that distinct bacterial groups emerged at different stages of fermentation (Figure 2). At the 8 h mark, the predominant enriched bacteria included members of Enterobacteriaceae, Gammaproteobacteria, *Klebsiella*, and *Escherichia-Shigella*, among others. By the 24 h point, the dominant species shifted towards Veillonellales-Selenomonadales, Selenomonadaceae, and *Megamonas*. At 36 h, the primary species included *Streptococcus*, among others, while at 72 h Firmicutes, *Mitsuokella*, and Veillonellaceae, along with *Megasphaera*, *Bifidobacterium*, Bifidobacteriaceae, Bifidobacteriales, and *Lactobacillus* genus, were prevalent. 

### 3.2. Alterations in AGE-Modified Polypeptides before and after Fermentation

**Figure 3:**
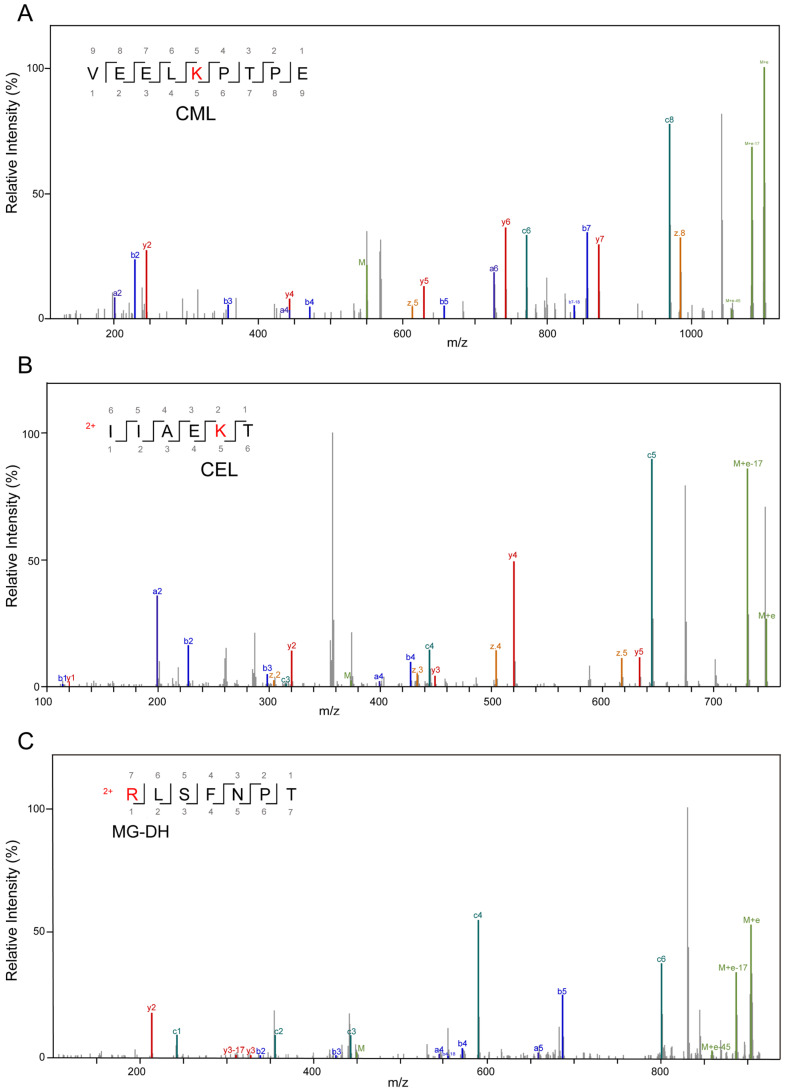

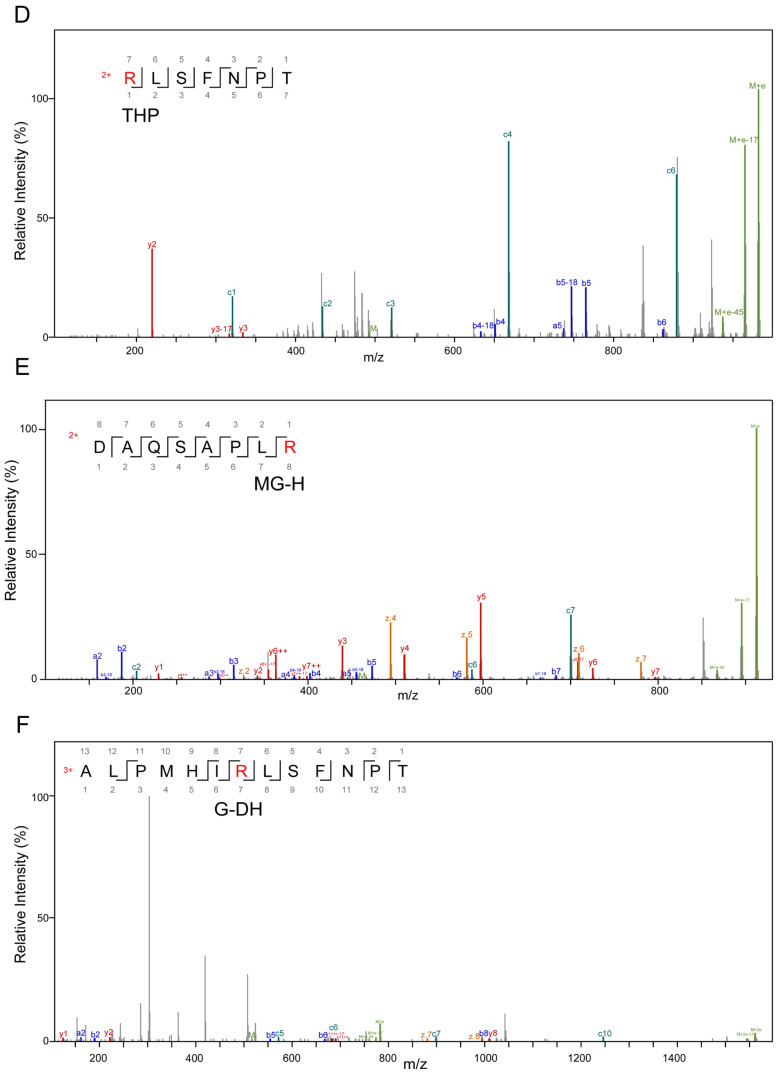

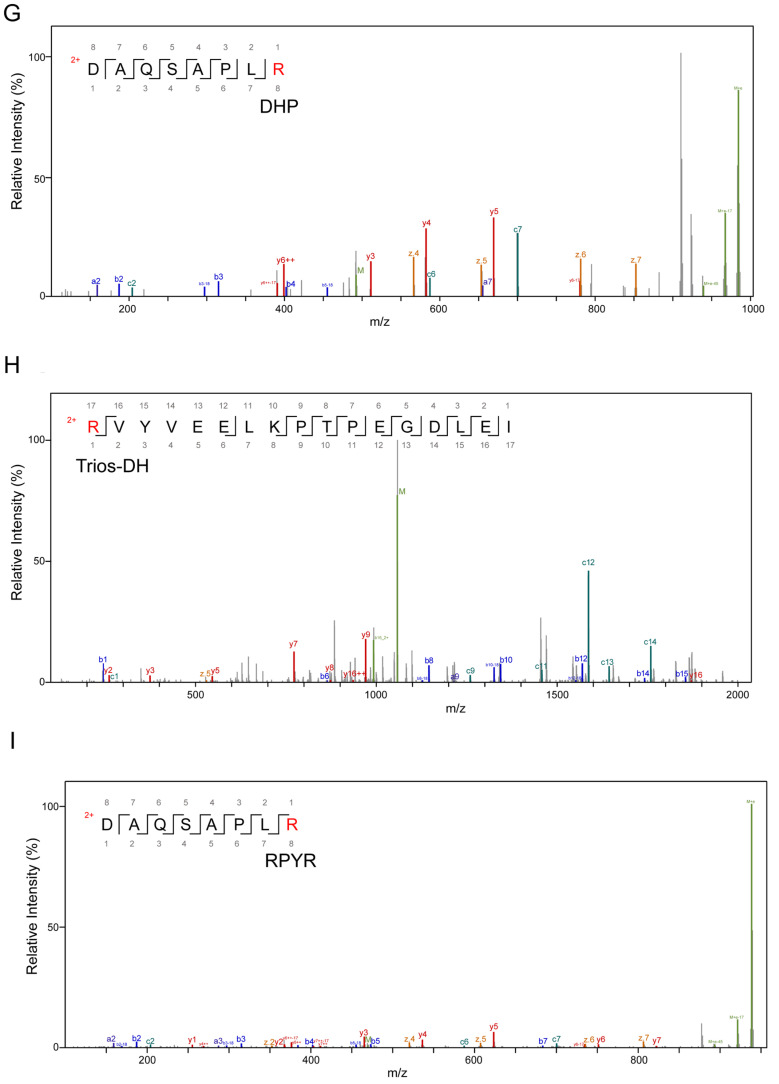
Secondary mass spectrum of AGE-modified peptides, different modification types, and modification sites. (**A**) Modification type of CML on lysine residues; (**B**) is a modification type of CEL on lysine residues; (**C**) is a modification type of MG-DH on arginine residues; (**D**) is a modification of THP on arginine residues; (**E**) is a modification type of MG-H on arginine residues; (**F**) is a modification type of G-DH on arginine residues; (**G**) is a modification type of DHP on arginine residues; (**H**) is a modification type of Trios-DH on arginine residues; (**I**) is a modification type of RPYR on arginine.

**Figure 4:**
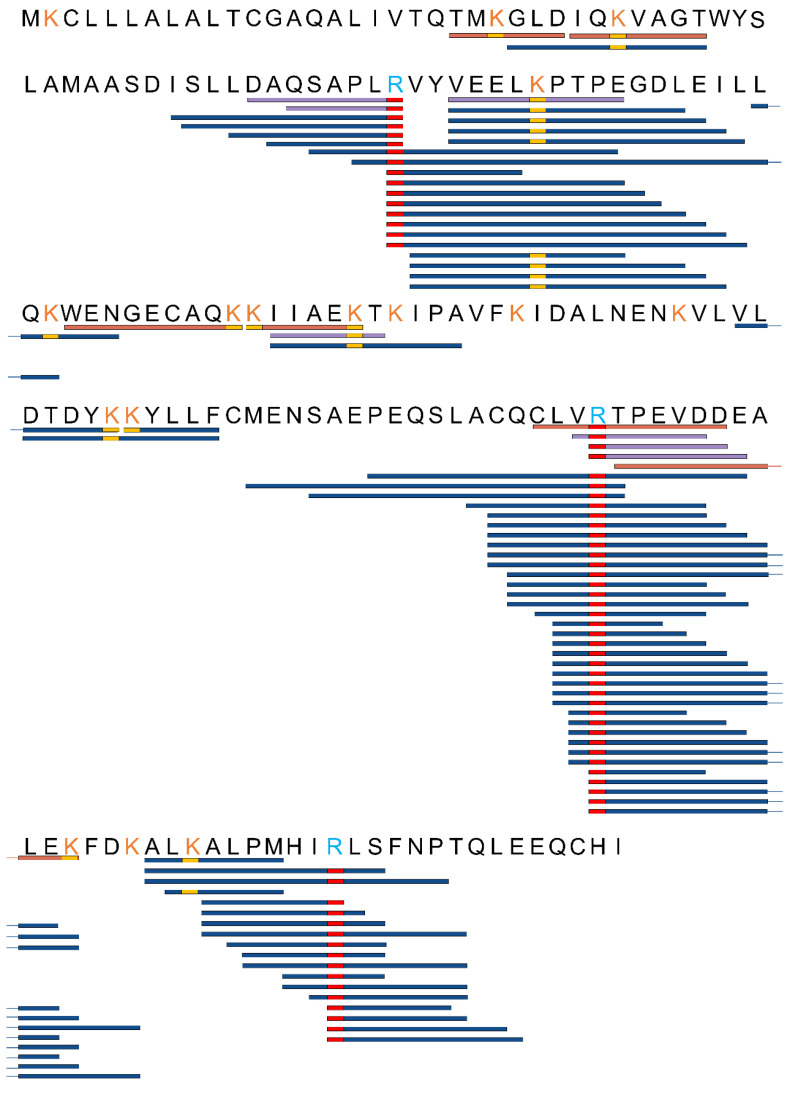
The sequences of polypeptides which had been subjected to AGEs modifications were compared both before and after the fermentation process. This comparison was made using high-resolution mass spectrometry. Peptides were categorized into three distinct groups based on their detection status: orange peptides, exclusively detected in samples before fermentation; blue peptides, exclusively detected in samples after fermentation; and purple peptides, which were detected both before and after fermentation. The yellow rectangle designates peptides with AGE modifications at lysine, while the red rectangle indicates peptides with AGE modifications at arginine.

High-resolution mass spectrometry was applied to detect modified peptide fragments in samples before and after fermentation. The detected AGE modifications encompassed nine distinct types, including CML, CEL, MG-DH, THP, MG-H, G-DH, DHP, Trios-DH, and RPYR, as illustrated in Figure 3. The alterations in peptides pre- and post-fermentation revealed that protein fragmentation resulted in the generation of peptides with varying lengths (Figure 4 and Table S2). Before fermentation, a total of 18 different types of AGE-modified peptides were identified, whereas after fermentation, the detection expanded to a total of 129 different types of AGE-modified peptides. This suggests that bacterial endonucleases might have hydrolyzed the AGE-binding protein, leading to increased diversity in the produced peptides. The observed significant increase in both the types and quantities of AGEs following fermentation suggests the catabolism of MGO-β-LG AGEs by human fecal microbiota.

### 3.3. Isolation and Screening of Potentially AGE-Metabolizing Bacteria from Fermentation Broth

**Figure 5:**
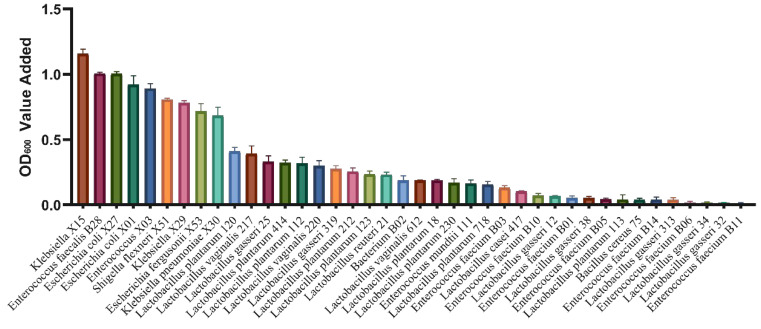
The OD values of bacteria post-cultivation were assessed using MGO-β-LG AGEs as the exclusive nitrogen source. Each distinctively colored column in the chart represents a unique bacterial strain.

A total of 187 bacterial strains were isolated (Table 2) at the early fermentation stage (8 h) and the late fermentation stage (72 h). Among these, at the 8 h fermentation stage, *Enterococcus* (*n* = 17), *Escherichia coli* (*n* = 17), *Klebsiella* (*n* = 3), *Bacterium* (*n* = 4), *Shigella flexneri* (*n* = 1), and *Escherichia fergusonii* (*n* = 1) were identified. At the 72 h fermentation stage, *Enterococcus* (*n* = 58), *Lactobacillus gasseri* (*n* = 57), *Lactobacillus plantarum* (*n* = 13), *Lactobacillus vaginalis* (*n* = 13), *Bacillus cereus* (*n* = 1), *Lactobacillus casei* (*n* = 1), and *Lactobacillus reuteri* (*n* = 1) were isolated. These isolated bacterial species basically covered most of the bacterial composition before and after fecal fermentation. Subsequently, individual strains were cultivated using MGO-β-LG AGEs as the sole nitrogen source. The fluctuations in optical density (OD) values were carefully monitored before and after cultivation to elucidate the distinctive capabilities of different strains in utilizing AGEs as a nitrogen source. The results illustrated in Figure 5 reveal that among the screened strains, one particular *Klebsiella* strain exhibited the most substantial change in OD, indicating the highest potential for AGEs metabolism. Consequently, this strain was selected as the representative bacterium for subsequent experiments.

### 3.4. Degradation of CML and CEL during Gut Microbiota and Klebsiella Fermentation

**Figure 6:**
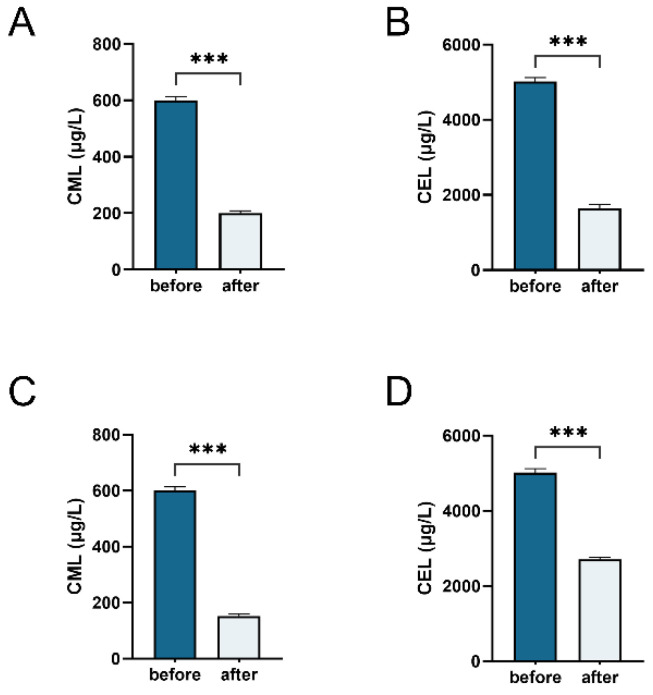
Determination of total amounts of CML and CEL. Measuring the total amounts of CML (**A**) and CEL (**B**) in fecal fermentation broth before and after fermentation. Total amounts of CML (**C**) and CEL (**D**) in the culture medium of *Klebsiella* X15 before and after cultivation. *** denotes significant differences between groups, with *p* < 0.001.

A reduction in CML and CEL content serves as a direct indicator of the metabolic breakdown or utilization of AGEs by the bacterial microbiota when MGO-β-LG AGEs are the sole nitrogen source. Liquid chromatography–mass spectrometry (LC-MS) was employed to monitor alterations in the total amount of CML and CEL during the fermentation process of human fecal samples (Figure 6A,B) and *Klebsiella* (Figure 6C,D). The results of bacterial fermentation reveal a noteworthy decrease in both CML and CEL contents post-fermentation. Similar outcomes were observed in *Klebsiella* X15 when MGO-β-LG AGEs was used as the sole nitrogen source. These findings affirm that *Klebsiella* X15 possesses the capability to degrade AGEs.

### 3.5. Proteomic Analysis of Klebsiella X15

**Figure 7:**
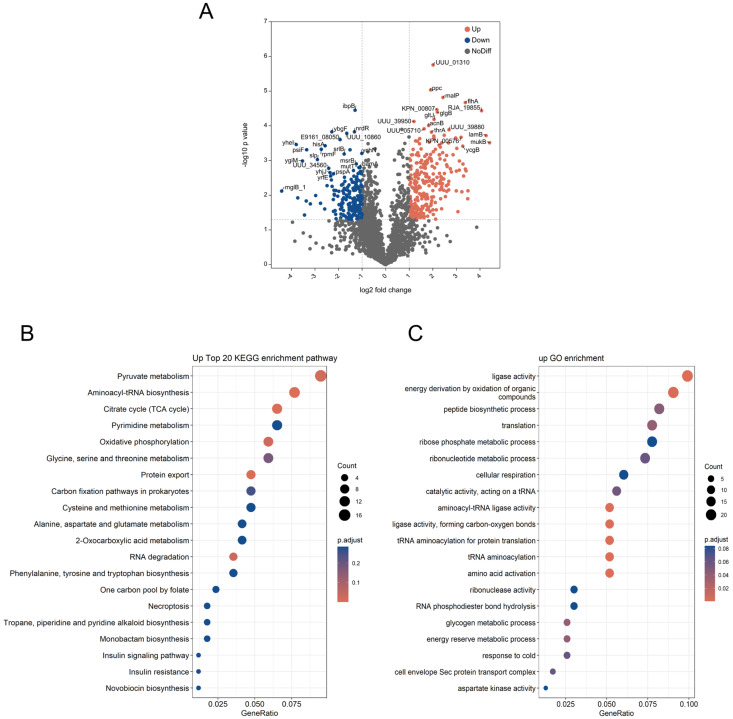

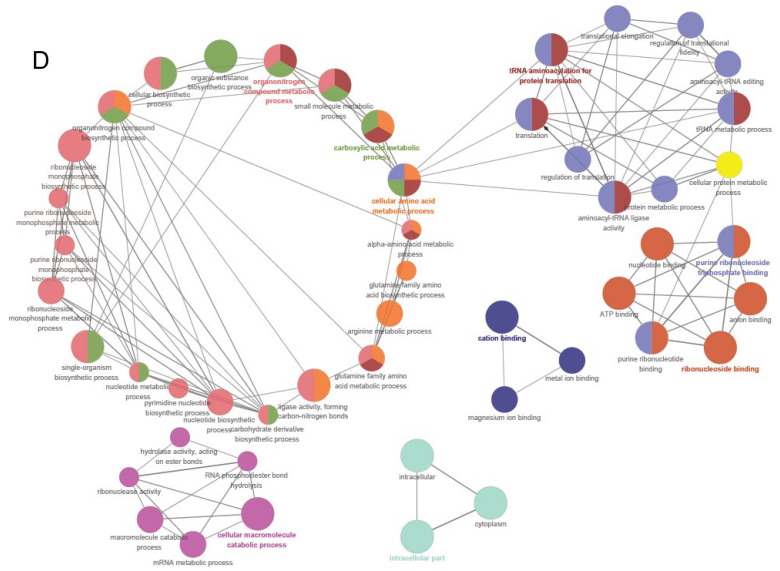
Proteomic analysis. Proteome volcano plot (**A**). In this plot, scattered points depict the differential fold changes of various proteins. The x-axis represents the log2 fold change, while the y-axis represents −log10 *p* values. The color blue signifies significant increases, and red represents significant decreases (*p* < 0.05). GO and KEGG enrichment analysis of different expressed proteins: GO enrichment analysis for molecular function, cellular component, and biological processes (**B**). The upregulated differentially expressed proteins from the KEGG pathway analysis (**C**). Protein–protein interaction (PPI) network (**D**). The PPI analysis was based on fold changes of protein expression, the PPIs, GO, and KEGG pathways, and biological process enrichments. Different circle colors represent different biological processes.

Label-free relative quantitative mass spectrometry was employed to investigate the changes in protein expression within *Klebsiella* X15 bacteria when MGO-β-LG AGEs were used as the sole nitrogen source, with unmodified protein serving as a control for comparative analysis. A total of 2131 proteins were identified. The volcano plot (Figure 7A) illustrates the distribution of differential proteins. Altogether, 254 proteins showed a significant increase, while 202 exhibited a notable decrease in MGO-β-LG AGE compared to the unmodified protein. Association with amino acid metabolism and synthesis revealed notable distinctions in amino acid metabolism within the context of MGO-β-LG AGEs, including a notable upregulation in the levels of aspartate ammonia-lyase, phosphoenolpyruvate carboxylase (PPC), aspartate aminotransferase family protein, glutamate/aspartate transport protein (ABC superfamily, membrane), peptide chain release factor 3, and protein translocase subunit SecD. 

To explore the biological significance of these differential proteins further, we conducted KEGG and GO enrichment analyses on the 254 proteins exhibiting increased expression (Figure 7B). The enriched KEGG pathways primarily include pyruvate metabolism; aminoacyl-tRNA biosynthesis; oxidative phosphorylation; protein export; cysteine and methionine metabolism; alanine, aspartate, and glutamate metabolism; and phenylalanine tyrosine and tryptophan biosynthesis; among others. The differentially expressed proteins were enriched in GO terms (Figure 7C). Notably, these proteins displayed heightened ligase activity, peptide biosynthetic process, aminoacyl-tRNA ligase activity, amino acid activation, energy reserve metabolic process, aspartate kinase activity, and so on. 

To further clarify functional correlations within these clusters, a network diagram of protein–protein interactions was derived through PPI analysis. The PPI network is categorized into 31 distinct protein clusters (Figure 7D). The orange clusters are characterized by larger circles and are associated with cellular amino acid metabolism, amide amino acid biosynthesis, carboxylic acid metabolism, arginine metabolism, alpha amino acid metabolism, and alpha-amino acid biosynthesis. Remarkably, when β-LG AGEs were the exclusive nitrogen source, the expression of proteins related to some amino acid metabolism levels experienced a significant upregulation. Furthermore, this upregulation also happened on tRNA aminoacylation in protein translation, ribonucleoside monophosphate biosynthesis, and the tricarboxylic acid (TCA) cycle. These observations suggest that bacteria employ an upregulation strategy for the aforementioned biological processes and signaling pathways when metabolizing MGO-β-LG AGEs. 

## 4. Discussion

Prior research has indicated that *Escherichia coli* can thrive in minimal media in the presence of CML [16], and one study has isolated an *Oscillibacter* from a culture medium utilizing free CML [17]. However, until now, no studies have employed protein-bound AGEs as the sole nitrogen source to isolate potential AGE-degrading bacteria. This study marks the first instance of using protein-bound AGEs as a fermentation substrate and isolating potential bacteria involved in metabolizing and decomposing these bound AGEs. 

The 8 h shift towards Proteobacteria suggests microbial characteristics associated with diseases, particularly metabolic disorders [22] and inflammatory bowel disease [23]. Previous studies have indicated that in the elderly, microbiota composition tends to exhibit reduced diversity [24], with Proteobacteria assuming dominance [25]. Throughout shifts in bacterial microbiota, special attention should be directed towards the predominant early-stage fermentation bacterium *Klebsiella*. What is noteworthy is its ubiquity in humans, livestock, plants, and soil, and its belonging to the Enterobacteriaceae family [26]. It is recognized as an opportunistic pathogen and one of the most common causes of nosocomial infections [27]. *Klebsiella* has also been associated with neonatal sepsis [28], frequently ranking among the top three pathogens in such cases. Its presence is often linked to cancer, as well [29]. 

Proteomics has emerged as a valuable tool for investigating diverse metabolic processes in bacteria [30,31]. Its reliability and innovative application in studying bacterial responses to different substrates are well-established. This innovative approach marks the first instance of employing proteomics to unravel the intricate ways in which bacteria interact with AGEs as a nitrogen source. Proteomics revealed a significant increase in aspartate ammonia-lyase expression. Phosphoenolpyruvate carboxylase (PPC) demonstrates upregulation specifically in response to AGEs. PPC is predominantly utilized for oxaloacetate synthesis during glucose metabolism, and in *E. coli*, which plays a key role in the anaerobic conversion of carbon into succinate. The heightened PPC activity in *E. coli* contributes to a reduction in both glucose consumption rate and the specific rate of ATP production, leading to a diminished overall cellular metabolic activity (manifested by a 37% decrease in ATP production) [32]. This observation highlights the restriction of bacterial growth when MGO-β-LG AGEs serve as the exclusive nitrogen source, a pattern that aligns with bacterial growth trends observed under oligotrophic conditions. Aspartate ammonia-lyase is a microbial enzyme that facilitates the reversible conversion of L-aspartate into fumarate and ammonia, which plays a crucial role in linking amino acid metabolism with organic acid metabolism. MGO-β-LG AGEs upregulated aspartate ammonia-lyase when employed as the exclusive nitrogen source. Research has revealed its ability to catalyze L-phenylalanine to generate trans-cinnamic acid in *Pseudomonas aeruginosa* [33]. The heterologous expression of aspartate ammonia-lyase from *Lactobacillus paracasei* in Escherichia coli has demonstrated its capability to catalyze aspartic acid, fumaric acid, phenylalanine, and tyrosine, resulting in the production of fumaric acid, aspartic acid, trans-cinnamic acid, and *p*-coumaric acid [34]. This wide substrate range suggests its potential to catalyze MGO-β-LG AGEs. Additionally, a family of aspartate aminotransferase has been implicated in nitrogen distribution in *Mycobacterium tuberculosis* [35]. Furthermore, the notable increase in the expression of multiple oxidoreductases indicates alterations in protein amino acid metabolism when MGO-β-LG AGEs are employed as substrates.

The significant abundance of differential proteins indicates potential alterations in bacterial metabolic pathways when MGO-β-LG AGEs serve as the sole nitrogen source. This implies that the metabolic processes associated with protein-bound AGEs differ from those involving general proteins. The exclusive use of MGO-β-LG AGEs as the sole nitrogen source may confer a competitive edge to *Klebsiella* X15 when AGEs are the exclusive nitrogen source. This study underscores the capacity of *Klebsiella* and other AGE-metabolizing bacteria in the intestine to metabolize bound AGEs, promoting their proliferation. These findings suggest a significant role for intestinal microbiota in mediating the impact of AGEs on human health.

## 5. Conclusions

This study employed MGO-β-LG AGEs as the exclusive nitrogen source for human gut microbiota fermentation to identify potential AGE-degrading bacteria. *Klebsiella* exhibited robust growth using MGO-β-LG AGEs as the sole nitrogen source, emphasizing its competitive advantage in AGE utilization. Proteomic analysis further highlighted increased proteins in *Klebsiella* related to amino acid and energy metabolism pathways during AGE utilization. This study is the first to isolate bacteria capable of metabolizing and decomposing protein bound AGEs. And it illuminates the pivotal role of gut microbiota, particularly *Klebsiella*, in metabolizing protein-bound AGEs within the intestine. This process influences microbial proliferation and contributes to a deeper understanding of the complex interplay between intestinal microbiota and AGEs.

## Figures and Tables

**Table 1:** Setting of AGE types.

Modification Sites	Types	Added MW (Da)	Added Composition
K	CML	58.01	C(2)O(2)H(2)
	CEL	72.02	C(3)O(2)H(4)
	PYR	108.02	C(6)O(2)H(4)
R	G-H	39.99	C(2)O(1)
	MG-H	54.01	C(3)O(1)H(2)
	G-DH	58.01	C(2)O(2)H(2)
	Trios-H	70.01	C(3)O(2)H(2)
	MG-DH	72.02	C(3)O(3)H(4)
	RPYR	80.03	C(5)O(1)H(4)
	Trios-DH	88.02	C(3)O(3)H(4)
	DHP	126.03	C(6)O(3)H(6)
	THP	144.04	C(6)O(4)H(8)

**Abbreviations:** CML, Nε-carboxymethyl-lysine; CEL, Nε-carboxyethyl-lysine; PYR, pyrraline; G-H, glyoxal-derived hydroimidazolone; MG-H, methylglyoxal-derived hydroimidazolone; G-DH, glyoxal-derived dihydroxyimidazoline; Trios-H, triosone-derived hydroimidazolone; MG-DH, methylglyoxal-derived dihydroxyimidazoline; RPYR, argpyrimidine; Trios-DH, triosone-derived dihydroxyimidazoline; DHP, N^δ^-(4-carboxy-4, 6-dimethyl-5-hydroxy-1, 4-di-hydropyrimidine-2-yl)ornithine; THP, N^δ^-(4-carboxy-4,6-dimethyl-5,6-dihydroxy-1,4,5,6-tetra-hydropyrimidine-2-yl)ornithine.

**Table 2:** Bacteria isolated fermentation broth at different time points.

Bacterial Genus and Species	No. of Isolates	Comments
*Lactobacillus gasseri*	57	From 72 h sample
*Lactobacillus plantarum*	13	From 72 h sample
*Lactobacillus vaginalis*	13	From 72 h sample
*Enterococcus*	75	From 8 h and 72 h sample
*Escherichia coli*	17	From 8 h sample
*Klebsiella*	3	From 8 h sample
*Bacterium* T15	4	From 8 h sample
*Bacillus cereus*	1	From 72 h sample
*Lactobacillus casei*	1	From 72 h sample
*Lactobacillus reuteri*	1	From 72 h sample
*Shigella flexneri*	1	From 8 h sample
*Escherichia fergusonii*	1	From 8 h sample

## Data Availability

The data presented in this study are openly available. The article has marked the location where the data can be accessed.

## Supplementary Materials

The following supporting information can be downloaded at: https://www.mdpi.com/article/10.3390/nu16050754/s1, Table S1, Composition of simulated gastric and intestinal digestion buffers. Table S2, Characteristic ions and MS/MS parameters of AGEs. Table S3, Types of modified peptides before and after fermentation.
