# Supplementary material for: The Interaction between Human Microbes and Advanced Glycation End Products: The Role of Klebsiella X15 on Advanced Glycation End Products’ Degradation
Source: Nutrients. 2024 Mar 6;16(5):754. doi: 10.3390/nu16050754 (PMC10933965; doi:10.3390/nu16050754)
Supplement: Supplementary file 1 [file nutrients-16-00754-s001.zip › nutrients-2904665-supplementary.pdf]

Supporting Information

The Interaction between Human Microbes and AGEs: Role of *Klebsiella* X15 on AGEs Degradation

Aiying Shi, Xuemeng Ji, Wanhua Li, Lu Dong, Yuekun Wu, Yunhui Zhang, Xiaoxia Liu, Huan Lv, Yan Zhang*, Shuo Wang

Tianjin Key Laboratory of Food Science and Health, School of Medicine, Nankai University, Tianjin 300071, China

*Corresponding authors: Yan Zhang

Tel: +86-22-85358730

Email: yzhang@nankai.edu.cn

MATERIALS AND METHODS

Gut microbiota analysis during *in vitro* fermentation. DNA extraction was conducted following the guidelines of the DNA extraction kit (E.Z.N.A.® Stool DNA kit, Omega Bio-tek, Norcross, GA, U.S.). The quality of the extracted DNA was assessed using agarose gel electrophoresis (1%), and the concentration of the extracted DNA was determined using NanoDrop2000. For PCR amplification of the 16S rRNA gene, the primers used were 27F (5'-AGRGTTYGATYMTGGCTCAG-3') and 1492R (5'-RGYTACCTTGTTACGACTT-3'). The reaction mixture consisted of 4 μL of 5×FastPfu buffer, 2 μL of 2.5 mM dNTPs, 0.8 μL each of the forward and reward primers (5 μM), 0.4 μL of FastPfu polymerase, 0.2 μL of BSA, 10 ng of template DNA, and ddH_2O to make up a total volume of 20 μL. The PCR program involved initial denaturation at 95 °C for 3 min, followed by 27 cycles of denaturation at 95 °C for 30 seconds, annealing at 60 °C for 30 seconds, and extension at 72 °C for 30 seconds, with a final extension step at 72 °C for 10 min. The PCR products were stored at 4 °C after completion. Following magnetic bead purification, quantification of the DNA was performed using the Quantus™ Fluorometer (Promega, USA). Library construction was carried out using the SMRTbell® Express Template Prep Kit 2.0, and sequencing was conducted using the Pacbio Sequel II System (Meiji Biomedical Technology Co., Ltd., Shanghai, China). Circular consensus sequences were generated using SMRTLink 8.0 software, and OTU clustering based on 97% similarity was performed using UPARSE 7.1 (http://drive5.com/uparse/, version 7.1). The sequences were then aligned and compared to the Silva 16S rRNA gene database (v138). Alpha diversity was

analyzed utilizing mothur software (http://www.mothur.org/wiki/Calculators), and differential bacteria at various time points were identified using LEfSe analysis (http://huttenhower.sph.harvard.edu/LEfSe) with $LDA>3.5$ and $p<0.05$.

PCR amplification. PCR amplification was conducted using Rapid Taq Master Mix, with a reaction mixture comprising 10 μL of the mix, 1 μL each of forward and reward primers (as previously described), 100 ng of template DNA, and ddH2O added to achieve a total volume of 20 μL. The PCR program involved an initial denaturation step at 95 °C for 3 min, followed by 30 cycles of denaturation at 95 °C for 30 seconds, annealing at 55 °C for 30 seconds, extension at 72 °C for 30 seconds, and a final extension step at 72 °C for 5 min, followed by incubation at 4 °C. Following PCR amplification, the sequence of the 16S rDNA fragment was determined using sequencing technology. Sequence data were subsequently analyzed, and a BLAST search was conducted against the NCBI database to identify the origin of the isolated bacterial strains. The remaining bacterial solution was mixed with an equal volume of 50% sterile glycerol solution and stored at -80 °C for future use.

Table S1 Composition of simulated gastric and intestinal digestion buffers

Reagent	Relative molecular mass	Concentration(M)	Concentration(g/L)	volume(mL)	quality(g)	Simulated digestion fluids for gastric		Simulated digestion fluids for intestinal	
						Concentration (mM)	volume (mL)	Concentration(mM)	Volume (mL)
KCl	74.55	0.5	37.3	100	3.73	6.9	6.9	6.8	6.8
KH_2PO_4	136.09	0.5	68	20	1.36	0.9	0.9	0.8	0.8
$NaHCO_3$	84.01	1	84	100	8.4	25	12.5	85	42.5
NaCl	58.44	2	117	100	11.7	47.2	11.8	38.4	9.6
$MgCl_2(H_2O)_6$	203.3	0.15	30.5	10	0.305	0.1	0.4	0.33	1.1
$(NH_4)_2CO_3$	96.09	0.5	48	10	0.48	0.5	0.5		
$CaCl_2(H_2O)_2$	147.01	0.3	44.1	100	4.41	0.15	0.005	0.6	0.04

Table S2 Characteristic ions and MS/MS parameters of AGEs

Compounds	Molecular formula	Parent (m/z)	Daughter (m/z)	Dwell (s)	Cone (V)	Collision (V)
CML	$C_8H_{16}N_2O_4$	205.00	84.01#	0.054	6	20
			129.94	0.054	6	19
CEL	$C_9H_{18}N_2O_4$	219.00	84.01#	0.054	16	18
	$C_8H_{16}N_2O_4$		130.84	0.054	16	8

* # represents quantitative ion;

** Precision for CML: LOD>0.030 ppb, LQD>0.109 ppb; Precision for CEL: LOD>0.012 ppb, LQD>0.103 ppb;

Table S3 Types of modified peptides before and after fermentation

Before fermentation

Modified peptides sequence	Products of modification
Q.TMK[+72.021]GLD.I	CEL
D.IQK[+72.021]VAGT.W	CEL
L.DAQSAPLR[+54.010].V	MG-H
L.DAQSAPLR[+126.032].V	DHP
L.DAQSAPLR[+72.021].V	MG-DH
A.QSAPLR[+54.010].V	MG-H
Y.VEELK[+72.021]PTPE.G	CEL
Y.VEELK[+58.005]PTPE.G	CML
K.WENGECAQK[+58.005].K	CML
K.K[+72.021]IIAEK.T	CEL
K.IIAEK[+72.021]T.K	CEL
Q.CLVR[+54.010]TPEVDD.E	MG-H
L.VR[+54.010]TPEVD.D	MG-H
V.R[+72.021]TPEVDD.E	MG-DH
V.R[+54.010]TPEVDD.E	MG-H
V.R[+54.010]TPEVDDE.A	MG-H
V.R[+72.021]TPEVDDE.A	MG-DH
R.TPEVDDEALEK[+72.021].F	CEL

After fermentation

Modified peptides sequence	Products of modification
K.GLDIQK[+72.021]VAGT.W	CEL
D.ISLLDAQSAPLR[+54.010].V	MG-H
I.SLLDAQSAPLR[+80.026].V	RPYR
I.SLLDAQSAPLR[+54.010].V	MG-H
L.LDAQSAPLR[+54.010].V	MG-H
L.DAQSAPLR[+80.026].V	RPYR
L.DAQSAPLR[+54.010].V	MG-H
D.AQSAPLR[+80.026].V	RPYR
A.QSAPLR[+80.026].V	RPYR
Q.SAPLR[+54.010]VYVEELKPTPE.G	MG-H
A.PLR[+54.010]VYVEELKPTPEGDLEILLQK.W	MG-H
L.R[+80.026]VYVEEL.K	RPYR
L.R[+80.026]VYVEELKPTPE.G	RPYR
L.R[+54.010]VYVEELKPTPE.G	MG-H

L.R[+80.026]VYVEELKPTPEG.D	RPYR
L.R[+80.026]VYVEELKPTPEGD.L	RPYR
L.R[+54.010]VYVEELKPTPEGDL.E	MG-H
L.R[+80.026]VYVEELKPTPEGDL.E	RPYR
L.R[+80.026]VYVEELKPTPEGDLE.I	RPYR
L.R[+54.010]VYVEELKPTPEGDLE.I	MG-H
L.R[+54.010]VYVEELKPTPEGDLEI.L	MG-H
L.R[+126.032]VYVEELKPTPEGDLEI.L	DHP
L.R[+80.026]VYVEELKPTPEGDLEI.L	RPYR
L.R[+88.016]VYVEELKPTPEGDLEI.L	Trios-DH
L.R[+54.010]VYVEELKPTPEGDLEIL.L	MG-H
L.R[+80.026]VYVEELKPTPEGDLEIL.L	RPYR
V.YVEELK[+72.021]PTPE.G	CEL
V.YVEELK[+72.021]PTPEGDL.E	CEL
V.YVEELK[+58.005]PTPEGDL.E	CML
V.YVEELK[+72.021]PTPEGDLE.I	CEL
V.YVEELK[+72.021]PTPEGDLEI.L	CEL
Y.VEELK[+72.021]PTPE.G	CEL
Y.VEELK[+72.021]PTPEGDL.E	CEL
Y.VEELK[+72.021]PTPEGDLE.I	CEL
Y.VEELK[+58.005]PTPEGDLEI.L	CEL
Y.VEELK[+72.021]PTPEGDLEI.L	CEL
Y.VEELK[+72.021]PTPEGDLEIL.L	CEL
L.LQK[+72.021]WEN.G	CEL
K.IIAEK[+72.021]T.K	CEL
K.IIAEKTK[+72.021]IPA.V	CEL
L.VLDTDYK[+72.021].K	CEL
L.DTDYK[+72.021]KYLLF.C	CEL
K.K[+72.021]YLLF.C	CEL
E.PEQSLACQCLVR[+58.005]TPEVDDE.A	G-DH
C.MENSAEPEQSLACQCLVR[+54.010]T.P	MG-H
N.SAEPEQSLACQCLVR[+54.010]T.P	MG-H
L.ACQCLVR[+80.026]TPEVD.D	RPYR
A.CQCLVR[+126.032]TPEVD.D	DHP
A.CQCLVR[+126.032]TPEVDD.E	DHP
A.CQCLVR[+126.032]TPEVDDE.A	DHP
A.CQCLVR[+126.032]TPEVDDEA.L	DHP
A.CQCLVR[+126.032]TPEVDDEALE.K	DHP
A.CQCLVR[+126.032]TPEVDDEALEK.F	DHP
C.QCLVR[+72.021]TPEVDDEALEK.F	MG-DH
C.QCLVR[+72.021]TPEVD.D	MG-DH
C.QCLVR[+72.021]TPEVDD.E	MG-DH

C.QCLVR[+72.021]TPEVDDE.A	MG-DH
Q.CLVR[+126.032]TPEVD.D	DHP
Q.CLVR[+54.010]TPEVD.D	MG-H
C.LVR[+80.026]TPE.V	RPYR
C.LVR[+54.010]TPE.V	MG-H
C.LVR[+54.010]TPEV.D	MG-H
C.LVR[+80.026]TPEVD.D	RPYR
C.LVR[+54.010]TPEVD.D	MG-H
C.LVR[+54.010]TPEVDD.E	MG-H
C.LVR[+80.026]TPEVDD.E	RPYR
C.LVR[+80.026]TPEVDDE.A	RPYR
C.LVR[+54.010]TPEVDDE.A	MG-H
C.LVR[+54.010]TPEVDDEA.L	MG-H
C.LVR[+80.026]TPEVDDEA.L	RPYR
C.LVR[+54.010]TPEVDDEALE.K	MG-H
C.LVR[+80.026]TPEVDDEALEK.F	RPYR
C.LVR[+54.010]TPEVDDEALEK.F	MG-H
C.LVR[+54.010]TPEVDDEALEKFDK.A	MG-H
L.VR[+54.010]TPEV.D	MG-H
L.VR[+80.026]TPEVD.D	RPYR
L.VR[+54.010]TPEVD.D	MG-H
L.VR[+54.010]TPEVDD.E	MG-H
L.VR[+80.026]TPEVDD.E	RPYR
L.VR[+54.010]TPEVDDE.A	MG-H
L.VR[+80.026]TPEVDDE.A	RPYR
L.VR[+54.010]TPEVDDEA.L	MG-H
L.VR[+80.026]TPEVDDEA.L	RPYR
L.VR[+54.010]TPEVDDEALE.K	MG-H
L.VR[+54.010]TPEVDDEALEK.F	MG-H
L.VR[+80.026]TPEVDDEALEK.F	RPYR
V.R[+80.026]TPEVD.D	RPYR
V.R[+54.010]TPEVDD.E	MG-H
V.R[+80.026]TPEVDD.E	RPYR
V.R[+54.010]TPEVDDE.A	MG-H
V.R[+80.026]TPEVDDE.A	RPYR
V.R[+54.010]TPEVDDEA.L	MG-H
V.R[+80.026]TPEVDDEA.L	RPYR
V.R[+54.010]TPEVDDEALE.K	MG-H
V.R[+54.010]TPEVDDEALEK.F	MG-H
V.R[+54.010]TPEVDDEALEKFDK.A	MG-H
K.ALK[+72.021]ALPM.H	CEL
K.ALPMHIR[+126.032].L	DHP

K.ALPMHIR[+54.010]L.S	MG-H
K.ALKALPMHIR[+54.010]LS.F	MG-H
K.ALPMHIR[+54.010]LS.F	MG-H
K.ALPMHIR[+80.026]LS.F	RPYR
K.ALKALPMHIR[+80.026]LSFNP.T	RPYR
A.LK[+72.021]ALPM.H	CEL
K.ALPMHIR[+80.026]LS.F	RPYR
K.ALPMHIR[+54.010]LS.F	MG-H
K.ALPMHIR[+58.005]LSFNPT.Q	G-DH
K.ALPMHIR[+54.010]LSFNPT.Q	MG-H
K.ALPMHIR[+80.026]LSFNPT.Q	RPYR
A.LPMHIR[+54.010]LS.F	MG-H
L.PMHIR[+80.026]LS.F	RPYR
L.PMHIR[+54.010]LS.F	MG-H
L.PMHIR[+54.010]LSFNPT.Q	MG-H
M.HIR[+80.026]LS.F	RPYR
M.HIR[+54.010]LSFNPT.Q	MG-H
M.HIR[+80.026]LSFNPT.Q	RPYR
H.IR[+54.010]LSFNPT.Q	MG-H
H.IR[+80.026]LSFNPT.Q	RPYR
I.R[+54.010]LSFNP.T	MG-H
I.R[+126.032]LSFNPT.Q	DHP
I.R[+80.026]LSFNPT.Q	RPYR
I.R[+144.042]LSFNPT.Q	THP
I.R[+72.021]LSFNPT.Q	MG-DH
I.R[+54.010]LSFNPT.Q	MG-H
I.R[+80.026]LSFNPT.Q	RPYR
I.R[+54.010]LSFNPTQL.E	MG-H
I.R[+80.026]LSFNPTQLE.E	RPYR
I.R[+54.010]LSFNPTQLE.E	MG-H
E.LDGEPTPKLEEVYVR[+58.005].L	G-DH
